# Nanostructured PbS-Doped Inorganic Film Synthesized by Sol-Gel Route

**DOI:** 10.3390/nano12173006

**Published:** 2022-08-30

**Authors:** Adrian Ionut Nicoara, Mihai Eftimie, Mihail Elisa, Ileana Cristina Vasiliu, Cristina Bartha, Monica Enculescu, Mihaela Filipescu, César Elosúa Aguado, Diego Lopez, Bogdan Alexandru Sava, Mihai Oane

**Affiliations:** 1 Faculty of Chemical Engineering and Biotechnologies, University Politehnica of Bucharest, 1 Gh. Polizu Str., 011061 Bucharest, Romania; 2 National Institute for R & D for Optoelectronics-INOE 2000, 409 Atomistilor Str., 077125 Magurele, Romania; 3 National Institute of Materials Physics, Atomistilor 405 A, 077125 Magurele, Romania; 4 National Institute of Laser, Plasma and Radiation Physics, 409 Atomistilor Str., 077125 Magurele, Romania; 5 Department of Electrical, Electronic and Communications Engineering, Public University of Navarra, E-31006 Pamplona, Spain; 6 Institute of Smart Cities (ISC), Public University of Navarra, E-31006 Pamplona, Spain

**Keywords:** sol-gel method, lead sulfide, quantum dots, quantum confinement, optical properties, alumino-silico-phosphate glass, composite material

## Abstract

IV-VI semiconductor quantum dots embedded into an inorganic matrix represent nanostructured composite materials with potential application in temperature sensor systems. This study explores the optical, structural, and morphological properties of a novel PbS quantum dots (QDs)-doped inorganic thin film belonging to the Al_2_O_3_-SiO_2_-P_2_O_5_ system. The film was synthesized by the sol-gel method, spin coating technique, starting from a precursor solution deposited on a glass substrate in a multilayer process, followed by drying of each deposited layer. Crystalline PbS QDs embedded in the inorganic vitreous host matrix formed a nanocomposite material. Specific investigations such as X-ray diffraction (XRD), optical absorbance in the ultraviolet (UV)-visible (Vis)-near infrared (NIR) domain, NIR luminescence, Raman spectroscopy, scanning electron microscopy–energy dispersive X-ray (SEM-EDX), and atomic force microscopy (AFM) were used to obtain a comprehensive characterization of the deposited film. The dimensions of the PbS nanocrystallite phase were corroborated by XRD, SEM-EDX, and AFM results. The luminescence band from 1400 nm follows the luminescence peak of the precursor solution and that of the dopant solution. The emission of the PbS-doped film in the NIR domain is a premise for potential application in temperature sensing systems.

## 1. Introduction

Semiconductor colloidal quantum dots (CQDs) have attracted vast scientific and technological interest throughout the past three decades due to the unique adjustment of their optoelectronic properties by variation in size and composition. However, the nanoscale size brings in a large surface-to-bulk volume ratio, where the external surfaces have a pronounced influence on the chemical stability and physical properties of the semiconductor [1,2,3]. Lead sulfide (PbS) is an important direct narrow gap semiconductor material with an approximate energy band gap of 0.4 eV at 300 K and a relatively large excitation Bohr radius of 18 nm what makes PbS very suitable for infrared detection applications. A new study based on PbS quantum dots synthesized by colloidal chemistry was reported [4] in connection with the influence of temperature (10–300 K) and excitation power on the luminescence features. A blue shift of the intensity peak was noticed with temperature increasing along with a broadening of the emission bands due to the interactions of the charge carriers with phonons. Recent results show that colloidal PbS QDs have been highlighted as a distinguished photonic material in nanotechnology, with applications in the next generation of electronic and photonic devices due to their intense size- dependent effects. PbS QDs exhibit nearly infrared light emission with size variation. PbS QD solutions and thin films with narrow fluorescence emission, high quantum yield, outstanding photo-stability and photoluminescence in the range 1000–1650 nm by 785 nm excitation have been reported [5]. IV-VI QDs with sizes between 2.7–7.6 nm, photoluminescence in the range 800–2000 nm. and decay kinetics in the time interval of 0.01–10 µs have been reported [6] as well as IV-VI QDs photoluminescence in the range 800–2000 nm, kinetic luminescence properties between 0.8 and 1.7 μm, resolution of 3 ns, and PbS QDs sizes between 3.4–8.4 nm [7]. Recently, a study was published relating to the double-tunable emission of colloidal PbS quantum dots in dependence on nanoparticle size and environmental temperature, due to the core/shell heterostructure, applied for temperature sensing systems [8]. The ability to tailor the optical properties by changing the particle size gives QDs materials the potential to solve many of the challenges of luminescence-based temperature sensors. Thus, PbS QDs in graphene oxide and reduced graphene oxide [9]; PbS QDs emission photoluminescence in the temperature range 4–300 K, emission domain 1000–1200 nm, collected by 514 nm excitation [10]; PbS QDs emission in the temperature range 100–300 K with PbS QDs between 3–6.5 nm; and emission at 800–1000 nm [11] and optical fiber luminescent temperature sensors based on QDs [12] were reported. Higher quantum efficiency due to an increase in oscillator strength gives QDs the potential to rival traditionally used rare-earth ions as optically active centers for thermal sensing. A step toward developing useful low-cost opto-sensing devices consists of immobilizing QDs in solid support structures. Optically transparent organic matrices having PbS QDs desirable for opto-sensing applications have been reported [13]. The temperature-dependent emission of PbS QDs has recently been noticed, thus opening new possibilities for optical temperature sensing devices, using PbS QDs that show photoluminescence between 850–950 nm and a temperature range of 10–310 K, with a QD size of about 2.5 nm [14]. An easy synthesis method to obtain silica-coated PbS nanocomposites with tunable size and optical properties was reported in [15], and PbS QDs embedded in the silicate matrix had photoluminescence at 440 nm and 605 nm. Size-dependent optical properties of colloidal PbS QDs were investigated by combining the QD absorbance spectra with detailed elemental analysis of the QD suspensions [16]. The photoluminescence dependence on the size of PbS QDs embedded in a complex silicate glass was shown based on the electron-hole/trap states of the QDs interacting with the defects from the interface between PbS QDs and the glass network [17]. The microstructural and optical properties of hierarchical eight-arm PbS QDs-based sensors for Pb^2+^ ions and infrared detection for optical and bio-sensing applications were also presented [18]. PbS colloidal QDs photodetectors working in the NIR (Near infrared) spectral range were recently proposed encompassing a wide variety of applications including optical fiber communications, spectroscopy, imaging, security, remote sensing, and metrology in several fields such as food inspection, agriculture, pharmacology, and biology. Thanks to their strong confinement, QDs have enhanced light–matter interactions providing unique optical properties such as increased optical absorption and emission as well as size adjustment. For these reasons, QDs have attracted a lot of attention for several optoelectronic devices, including light emitting diodes, lasers, solar cells, photodetectors, and recording of optical information [19,20,21,22]. PbS thin films have been prepared by various techniques such as chemical deposition [23], spray pyrolysis [24], chemical bath deposition [25,26,27,28,29,30,31], electrodeposition [32], photo-accelerated chemical deposition [33], microwave heating [34,35], and the spin coating method [36].

Small-size PbSe/PbS Core/Shell CQDs have been investigated by Yanover et al. [37]. Thus, the core diameter ranges between 2 and 2.5 nm, the shell diameter between 0.5 and 1 nm, and the emission about 0.15 eV. Recently, small-size colloidal quantum dots (QDs) consisting of IV–VI semiconductors with PbSe/PbS core/shell structure were synthesized by a specially developed wet chemistry method. Their electronic properties were found by comparison of theoretical calculations with continuous wave and transient photoluminescence measurements at various temperatures [38]. The influence of interfacial strain on the optical properties of PbSe/PbS CQDs was studied. The derived strain profile was incorporated into a band structure calculation to evaluate the influence on the electronic band edges of the core/shell CQDs [39].

In this study, a new nanocomposite material based on PbS QDs-doped film belonging to the Al_2_O_3_-SiO_2_-P_2_O_5_ system was synthesized by the sol-gel method and thespin-coating technique. The sol-gel method applied in this study is appropriate for synthesizing PbS QDs-doped alumino-silico-phosphate thin films having optical properties. This method allows control of the composition of the doped thin film, ensuring the uniformity of the film thickness and chemical homogeneity of the deposited film due to the molecular-level mixing and processing of the precursors at relatively low temperatures. Moreover, the sol-gel method is a suitable method for producing a low-cost QDs-doped thin film. This method does not limit the choice of the substrate material, providing a very good method for exploring the optical properties of semiconductors [40].

To our knowledge, this nanostructured material with complex composition based on PbS QDs-doped inorganic film, synthesized in the present study, is reported for the first time in the specialty literature. This paper aims to study the correlation between optical, structural, and morphological properties of this composite material, consisting of a PbS crystalline phase and an amorphous oxide network. The dimension of the PbS nanoparticles was calculated by different routes and compared to the PbS quantum dots size from the starting solution. The authors consider that this nanocomposite material presents real scientific interest, and it is recommended as a good candidate for temperature-sensing systems.

## 2. Materials and Methods

### 2.1. Synthesis of the Film

The PbS QDs-doped film prepared in this study using the sol-gel method, spin coating technique, belongs to the Al_2_O_3_-SiO_2_-P_2_O_5_ system. The following chemical reagents were used as precursors: AlAcAc-aluminum acetylacetonate (C_15_H_21_O_6_Al), TEOS-tetraethoxysilane (Si(OC_2_H_5_)_4_), and TEP-triethylphosphate- (C_6_H_15_O_4_P). Other chemical reagents were: EtOH-ethanol (C_2_H_5_OH) as the reaction medium and MEA-monoethanolamine (C_2_H_7_NO) to catalyze the gelification process, namely, the hydrolysis and condensation reactions. The dopant used was PbS QDs, covered by oleic acid and dispersed in toluene, concentration 10 mg/mL, having an emission at 1400 nm. All the chemical reagents were of analytical grade and were bought from the standard producer Sigma-Aldrich, St. Louis, MO, USA. The molar ratios of the precursors were: TEOS/TEP = 2.5; TEOS/AcAcAl = 4; TEP/AcAcAl = 1.5, TEOS/EtOH = 2.6 × 10^−4^, and MEA/TEOS = 1.02. The final solution with pH = 8 was prepared using 5.9895 mL of precursor solution composed of TEOS, TEP, EtOH, and MEA and 5 mL of PbS solution. The solution was kept at room temperature, under continuous magnetic stirring, for 2 h, to achieve an improved homogenization of the starting reagents, followed by the deposition process by spin coating on a glass substrate [41,42]. The deposition process took place at 2000 rpm, for 20 s, 50 layers, each layer being dried on an electrical plate at 150 °C, for 2 min, to remove EtOH and water from the mixture and to promote the vitreous host matrix formation. The vacuum environment protects the PbS against oxidation, which could alter the luminescence of the film. The vitreous matrix is chemically and thermally stable, having a chemical composition that is capable of preventing, also, the oxidation of the dopant to get a stabilized emission [43].

An un-doped film was also synthesized, keeping the same molar ratios of the pre-cursors and the same preparation parameters as for the PbS-doped film.

### 2.2. Measurements

The deposition of the film was performed with a spin coater (WS-650SZ, Laurel Spinner, Laurell Technologies Corporation, North Wales, PA, USA) on a glass substrate of 2.5 × 2.5 mm^2^, which was chemically cleaned before the deposition process.

The X-ray diffraction spectrum was recorded with a Bruker D8 Advance device (CuKα = 1.5406 Å) Billerica, MA, USA, having a measurement error of ±0.1%, at room temperature, in the range of 10–70°. The scan was performed with a step of 0.05° and a step time of 10 s. Phase identification was made with ICDD Powder Diffraction Files database [44].

The optical absorption was measured using a spectrophotometer Lambda 1050, (PerkinElmer, Waltham, MA, USA) in the UV-Vis-NIR domain in the range of 320–2500 nm, with a measurement error of ±0.03%.

The luminescence spectra were collected with a spectrofluorometer FluoroLog-3, HORIBA Jobin Yvone S.A.S. (Paris, France) in the range of 850–1600 nm, using 850 nm excitation wavelength from a Xe lamp of 450 W, with a measurement error of ±0.5 nm.

Raman spectra were collected by means of an LabRam HR Evolution HORIBA, (Palaiseau, France), acquisition time 2 s, accumulation 20, laser 514 nm, hole diameter 100 micro, objective 50×, grating 600 gr/mm, ND filter 100%, range 100–16,000 cm^−1^, with a measurement error of ±0.5 cm^−1^.

The AFM images were acquired with an atomic force microscope (AFM), XE100 type from Park System Company (Suwon, Korea). Different areas such as 40 μm × 40 μm and 2 μm × 2 μm were scanned. The measurement error was ± 5%.

The morphology and elemental compositions of the samples were studied with a Carl Zeiss Gemini 500 Field Emission Scanning Electron Microscope FESEM (Carl Zeiss, Oberkochen, Germany) equipped with a Bruker (Bruker, Bremen, Germany) Quantax Energy dispersive X-ray spectrometer (EDS) with an energy resolution of 129 eV and Peltier cooling. Top- view FESEM images were evaluated without metallic coverage of the samples. The cross-section evaluation and the thickness of the films’ measurements were performed on freshly cleaved samples. The measurement error of the film thickness was ±1%, and for the EDS the measurement error was ±0.02%.

## 3. Results and Discussion

### 3.1. X-ray Diffraction Analysis (XRD)

**Figure 1:**
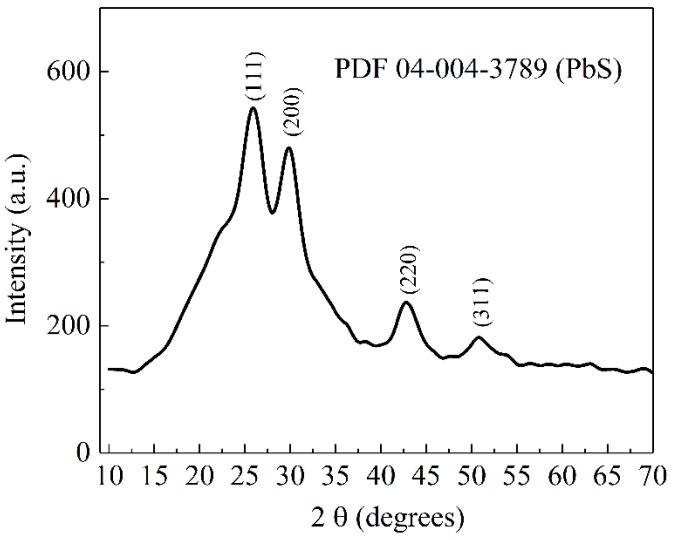
XRD pattern of PbS-doped film.

An XRD pattern of PbS-doped film is presented in Figure 1. It confirms the amorphous state of the host inorganic matrix (Al_2_O_3_-SiO_2_-P_2_O_5_), shown by a broad aspect of the spectrum. Distinct peaks corresponding to the PbS hexagonal crystalline phase (ASTM file-04-004-3789), localized on the amorphous halo, are noticed.

The nanocrystal size of PbS has been calculated using the Scherrer formula [41,45], Equation (1), applied to the main peaks found at 2*θ* = 25.8° (111), 29.77° (200) and 42.90° (220) respectively,

(1) $$d=\frac{k\lambda }{\Delta cos\theta }$$

where *k* is the Scherrer constant (the shape factor has a typical value of about 0.9); *λ*, the wavelength of the incident X-ray beam (*λ_CuKα_*_1_ = 1.5406 Å); *2θ*, the peak position of the reflection; and Δ, the full width at half maximum of the reflection. According to this calculation, the PbS grain size was estimated to be around 7.99 nm compared to the size of PbS QDs from the dopant solution in toluene about 7 nm (Sigma-Aldrich-Technical Specification).

### 3.2. Optical Properties

#### 3.2.1. Optical Absorption

**Figure 2:**
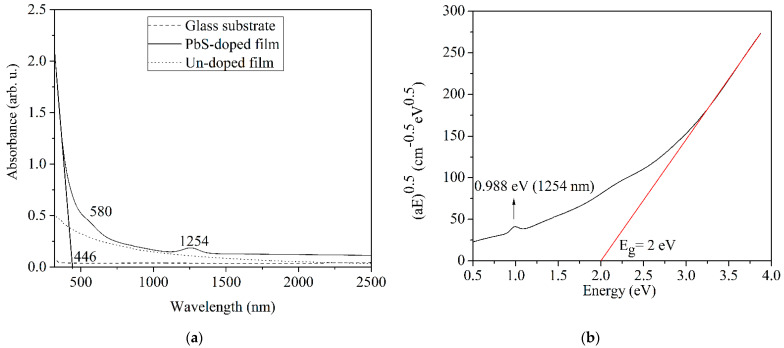
(**a**) Optical absorption of PbS-doped and un-doped film, and glass substrate; (**b**) graphical determination of the optical band gap value.

In Figure 2a, the optical absorption of the glass substrate and the PbS-doped film is presented. Extremely low absorption of the glass substrate is revealed, and a decrease in the optical absorption of the PbS-doped film is found from the UV to the visible domain, being almost constant in the NIR domain. An absorption peak is found at 1254 nm corresponding to the first exciton transition, and a low intense absorption peak is noticed at about 580 nm, corresponding to the second exciton transition.

The cutoff wavelength deduced from Figure 2a, is *λ_cutt-off_* = 446 nm.

For the un-doped film, we also revealed a decrease in the optical absorption from UV to the visible domain, being almost constant in the NIR domain. The optical absorption values are lower than in PbS-doped film, mainly in the UV and visible domain, without peaks, as in the case of the PbS-doped film.

The energy band gap, *E_g_*, of PbS QDs can be obtained as the sum of the exciton binding energy and the first exciton peak energy deduced from the absorption spectrum. It is approximated that the maximum exciton binding energy is four times the exciton binding energy in bulk materials, *E_bulk_* [46]. In the case of PbS, the exciton binding energy *E_bulk_* (PbS) is 3.968 meV [47]. Thus, for an electron effective mass of 0.085_m0_ (_m0_ is the free electron mass), equal to hole effective mass [48,49], and a dielectric constant of 17.5 [50], the maximum exciton binding energy of QDs is 15.872 meV. The first exciton peak energy deduced from the absorption spectrum is 0.988 eV and, consequently, the energy band gap, *E_g_* is 1.004 eV.

According to [41,43,51], the energy band gap dependency on the size of semiconductor QDs is approximated using the Equation (2):

(2) $$E_{g}^{QDs}=E_{g}^{Bulk}+\frac{h^{2}}{2m^{*\;}d^{2}}$$

(3) $$m^{*}=\frac{m_{1}m_{2}}{m_{1}+m_{2}}$$

where *E_g_^QDs^* is the effective band gap of PbS nanoparticles, *E_g_^Bulk^* is the band gap of PbS bulk, i.e., 0.4 eV [1], m* is the reduced mass of exciton [51], *m*_1_ is the effective mass of electron, *m*_2_ is the effective mass of hole, *m*_0_ is the free electron mass (Equation (3)), *h* is Planck’s constant, and *d* is the diameter of the PbS nanocrystals. The PbS nanoparticle size, *d*, determined from Equation (2), based on Equation (3), using *E_g_* = 1.004 eV, is about 7.72 nm, close to the value determined by XRD analysis, 7.99 nm. Thus, the quantum confinement effect, which is the dependency of the band gap value on the quantum dots size, is valid in the case of PbS-doped film, because the nanoparticle size is lower than the Bohr radius of the exciton in PbS, 18 nm [46,47].

The graphical determination of *E_g_* for the PbS-doped film is reported in [40,42,52]. The Mott and Davis/Tauc equation (4) was used to graphically establish the optical band gap, *E_g_* (Figure 2b). Thus, the Equation (4) [41,43,52]

(4) $$\alpha h\nu ={\left(h\nu -E_{g}\right)}^{n}$$

enables to determine the band gap, *E_g_*, where *α* is the absorption coefficient in dependence on wavelength, *h* is the Planck constant, *ν* is the light frequency, and *n* can be ½ for the allowed direct electron transition and 2 for the allowed indirect electron transition from the valence to the conduction band [53].

The absorption coefficient, *α*, is calculated using Equation (5) [54], as following:

(5) $$\alpha =\frac{A\times 2.303}{x}$$

where *A* is the optical absorbance in dependence on wavelength as it is presented in Figure 2a and *x* is the thickness of the film, 2.5 µm, as it was found from SEM analysis in cross section. In the case of PbS-doped film, the absorption coefficient, *α*, calculated using Equation (5) ranges between 1038–19,341 cm^−1^.

For amorphous materials, in the case of PbS-doped film, *n* takes the value of 2. The optical band gap value, *E_g_*, was obtained by extrapolating the linear region of the curve to the zero absorption at which αhν = 0, and the result was *E_g_* = 2 eV (see Figure 2b). The wavelength corresponding to the band gap value is *λ_g_* = 620 nm. Using *E_g_* = 2 eV and applying Equation (2), we calculated the nanoparticle size, *d* = 4.72 nm, which is relatively close to the values determined by XRD analysis and to the maximum exciton energy, respectively.

The difference in the bandgap value deduced from the first exciton energy, 1.004 eV and from Mott and Davis/Tauc’s law, 2 eV, could be explained considering the nature of the PbS-doped film. Thus, the synthesized film is a composite material consisting of an amorphous inorganic matrix (Al_2_O_3_-SiO_2_-P_2_O_5_) and crystalline nanoparticles of PbS quantum dots as shown by XRD analysis. The graphical determination of *E_g_* using Mott and Davis/Tauc’s law considered only the amorphous inorganic host matrix, taking *n* = 2. It did not take into consideration the crystalline phase consisting of PbS nanoparticles corresponding to *n* = ½. In fact, for such composite materials, the *n* value is not exactly known, as it depends on the nature and amounts of those two phases.

**Figure 3:**
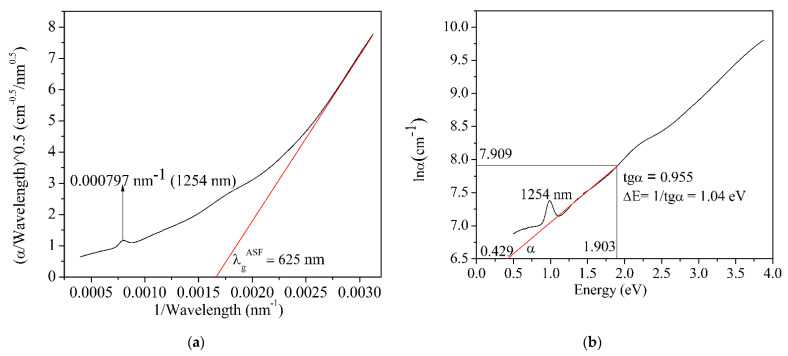
(**a**) variation of (*α/λ*)^0.5^ with 1/*λ*; (**b**) Urbach plot for PbS-doped glass.

To verify the validity of the graphical method to determine *E_g_* and, respectively, *λ_g_*, the absorption spectrum fitting (ASF) method was applied [41,43,52] by means of which $E_{opt}^{ASF}$ was determined and compared to the *E_g_* value determined by the Mott and Davis/Tauc law. Thus, the Tauc law is written as Equation (6) [41,43,52]:

(6) $$\frac{\alpha hc}{\lambda }={\left(\frac{hc}{\lambda }-E_{g}\right)}^{2}\;$$

where *α* is the absorption coefficient, *h* is the Planck constant, *c* is the speed of light in vacuum equal to 3 × 10^8^ m/s, *λ* is the wavelength, and *E_g_* is the band gap energy. Thus, the function ${\left(\alpha /\lambda \right)}^{0.5}=f\left(\frac{1}{\lambda }\right)$ is graphically represented in Figure 3a. The result is a curve, and the tangent to the linear region of the curve will intersect the x-axis in a point, 1/*λ_g(ASF)_*, resulting in *λ_g(ASF)_*.

This value is compared with that found from the Tauc law—in our case, 1/*λ_g(ASF)_* = 0.0016 and *λ_g(ASF)_* = 625 nm, which is remarkably close to that determined from the Tauc law, *λ_g_* = 620 nm. The Equation (7) [41,43,52]

(7) $$E_{opt}^{ASF}=\frac{1240}{\lambda_{g}}$$

is applied to calculate $E_{opt}^{ASF}$, which, in our case is 1.984 eV; this is remarkably close to 2 eV, which is graphically determined by the Tauc law.

Generally, for amorphous materials, the allowed indirect electron transitions according to the Tauc equation are valid.

In the case of optical absorption, for low photon energy ranging between 10^2^ and 10^4^ cm^−1^, the absorption coefficient follows Urbach’s law. This is the width of the band tails of the localized states from the valence band. Urbach’s law is depicted by the following Equation (8) [18,21,30]:

(8) $$\alpha \left(\vartheta \right)=\alpha_{0}\exp\left(\frac{h\vartheta }{\Delta E}\right)$$

where *α(ν)* is the absorption coefficient in dependency on the frequency, *hν* is the energy, and Δ*E* is the Urbach energy. If the logarithm of Urbach’s equation is applied, the following Equation (9) is obtained [41,43,52]:

(9) $$ln\alpha \left(\nu \right)=C+\frac{h\nu }{\Delta E}$$

Hence, it is possible to deduce the Δ*E* value, where *C* is a constant. The dependence of lnα on energy results in a curve, and the tangent to the linear region of the curve will intersect the x-axis in a point, corresponding to an energy value of 0.429. Taking into account the values of the projects of the tangent upper point on the x and y-axes, tgα was calculated. Thus, Urbach energy, Δ*E* was calculated.

In the case of PbS-doped film, Urbach energy, Δ*E*, is 1.04 eV (Figure 3b). Urbach energy is a measure of the disorder degree of the materials, and it is correlated with an extension of the localized states within the band gap [52]. In the case of PbS-doped film, Urbach energy value reveals a certain degree of disorder taking into consideration the composite material consisting of two phases, namely, the amorphous host matrix and the crystalline nanoparticles of the PbS dopant.

#### 3.2.2. Optical Emission

**Figure 4:**
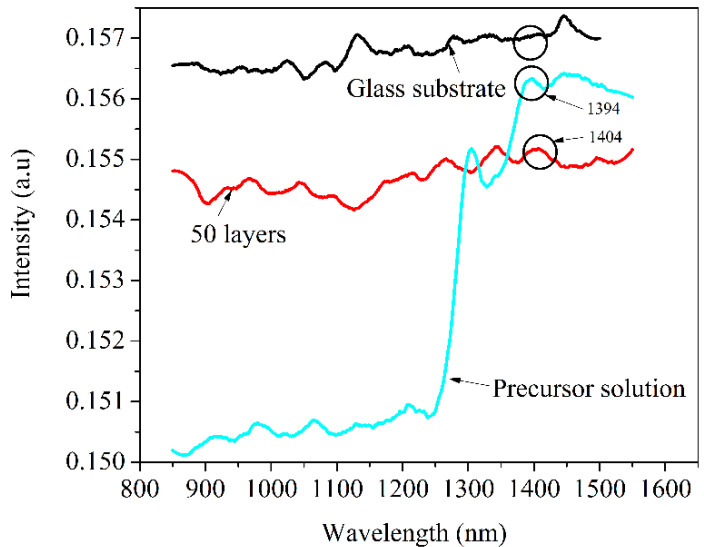
Optical emission of PbS-doped film, precursor solution and glass substrate, collected by 800 nm excitation.

The optical emissions of the glass substrate, PbS QDs dopant solution, and PbS-doped film are presented in Figure 4, collected at 800 nm excitation in the range of 850–1550 nm. We note a lack of emission at about 1400 nm in the case of the glass substrate as compared to the PbS-doped film where an emission peak is noticed around 1404 nm. The PbS QDs precursor dopant solution dispersed in toluene shows an emission maximum at about 1394 nm. The position of the photoluminescence peak in the case of PbS-doped film is slightly shifted toward higher wavelength, possibly due to the influence of the inorganic host matrix.

The electron/hole trap states of PbS QDs and the defect states at the interface between PbS QDs and the inorganic matrix have an important effect on the photoluminescence properties of PbS QDs.

These effects suggest that the surface trap states and defect states are strongly dependent on the size of the quantum dots [17]. When PbS QDs are photo-excited by 800 nm, a transition appears between 1S_h_ (hole ground state from the valence band, characterized by energy E_h0_) and 1S_e_ (electron ground state from the conduction band, characterized by energy E_v0_), corresponding to the first exciton peak found at 1254 nm from the absorption spectrum. Due to the quantum confinement effect, in the case of QDs with a size lower than the Bohr exciton radius (18 nm in the case of PbS), the energy levels of the hole states from the valence band and the energy levels of the electron states from the conduction band are quantized. The photo-generated electron will be trapped by the electron trap states (ETS) located below the 1S_e_ level, at high energy levels of the defect states (DS). Thus, a radiative transition occurs to the 1S_h_ level, corresponding to the peak found at 1404 nm in the case of PbS-doped film and 1394 nm in the case of the precursor solution. When the QDs size is increasing, according to Equation (2), the band gap value is decreasing, and ETS is located very close or overlapped by the level 1S_e_, both located at low energy levels from the DS. In this case, the radiative transition to the 1S_h_ level will occur, but the corresponding energy will decrease. Consequently, the emission wavelength will shift toward high values. The DS are present in the inorganic host matrix such as non-bridging oxygen atoms, structural modifiers, and other disordered structures situated at the interface with PbS QDs [17,55].

### 3.3. Raman Spectroscopy

**Figure 5:**
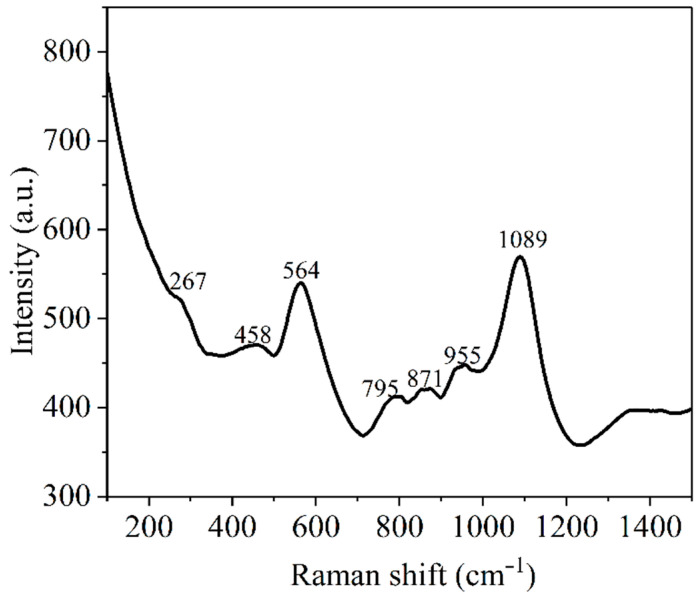
Raman spectrum of PbS-doped film, collected by 514 nm excitation.

In Figure 5, the Raman spectrum of PbS-doped film is presented, in the range of 100–1500 cm^−1^. Raman peaks could be assigned to the PbS dopant as well as to the Si-O and P-O vibration modes.

Thus, the peak at 267 cm^−1^ is assigned to PbS molecules [56,57], the peak at 458 cm^−1^ is assigned to σ and δ(Si-O-Si) [58], and the peak at 564 cm^−1^ of high intensity is assigned to γ(Si-O-Si) [59]. Low-intensity peaks are noticed at 795 cm^−1^, being assigned to σ_sym_(Si-O-Si) [60], σ(Si-O-R) and TEOS [61]; 871 cm^−1^ is assigned to σ(SiO_4_)^4−^ [60], and 955 cm^−1^ is assigned to σ(Si-O^−^) [59]. A high-intensity band is noticed at 1089 cm^−1^ that can be assigned to σ_asym_(Si-O-Si) [59], σ_asym_(SiO_4_)^4−^ [60] and σ(Si-O-P) [61]. The codes of the vibration modes are: σ = stretching vibration mode, δ = bending vibration mode, and γ = rocking vibration mode.

### 3.4. Scanning Electron Microscopy-Energy Dispersive X-ray (SEM-EDX)

**Figure 6:**
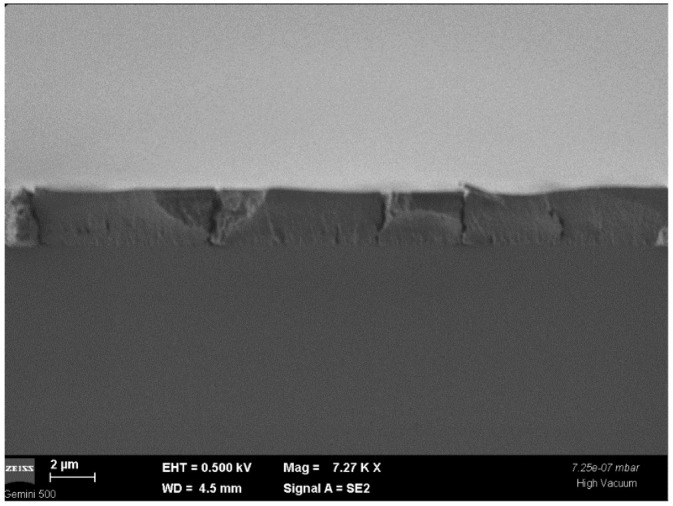
SEM image in a cross section of PbS-doped film, deposited on a glass substrate.

In Figure 6, an SEM image in a cross section of the PbS-doped film is presented. It is seen to be a relative uniform film with an evaluated average thickness of 2.5 µm. There are seen some cracks in the film due to the drying process, accompanied by water and alcohol releasing from the network.

**Figure 7:**
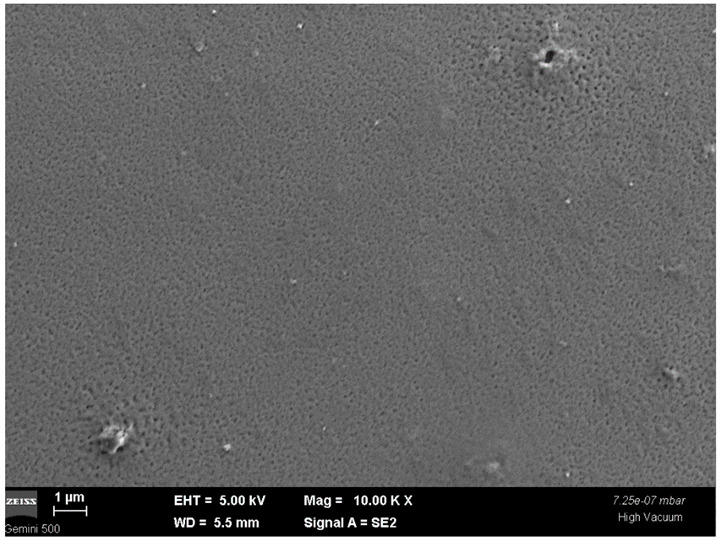
SEM image of the PbS-doped film surface.

In Figure 7, an SEM image of the PbS-doped film surface at 10,000 magnification is presented. One can see the film pores of tens of nanometers order that are specific to the sol-gel method.

**Figure 8:**
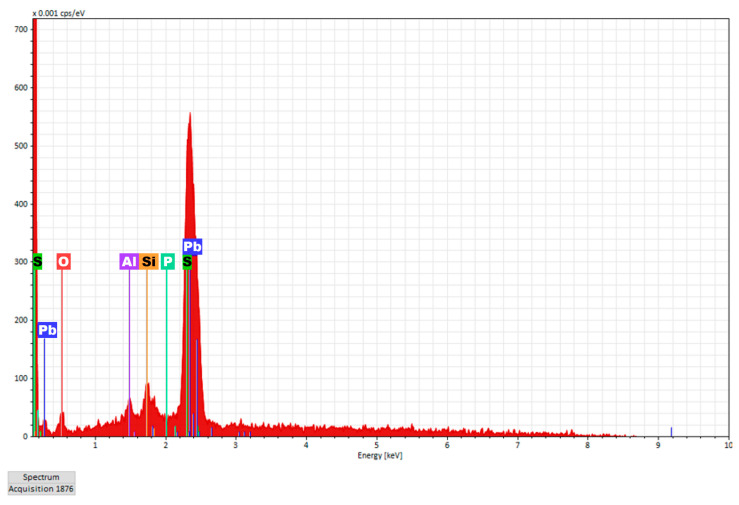
EDX elemental composition of the Pb-doped film deposited on glass substrate.

Figure 8 shows the EDX elemental composition of the Pb-doped film deposited on the glass substrate.

It is noticed that specific elements to both the film and the glass substrate are found, such as: Si and O, together with elements specific to the deposited film such as Al, P, Pb, and S.

### 3.5. Atomic Force Microscopy (AFM)

**Figure 9:**
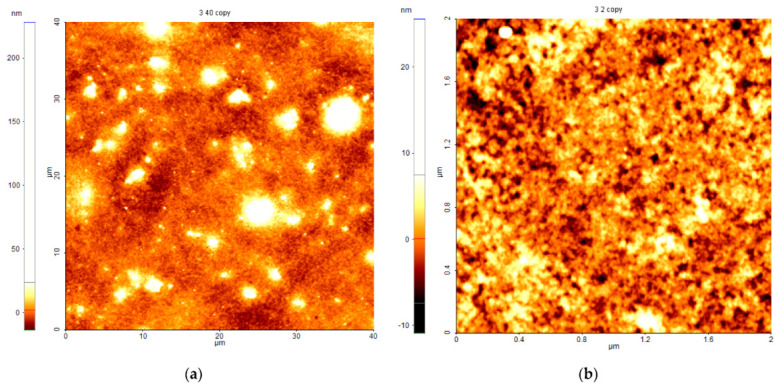
AFM images of the surface layer of the PbS-doped film, scanned on different areas: (**a**) 40 μm × 40 μm and (**b**) 2 μm × 2 μm.

The AFM images (Figure 9) were obtained by scanning different areas (40 μm × 40 μm and 2 μm × 2 μm). The AFM parameters revealed important data about the features of the layers and the roughness of the surface. The average squared roughness value, *Rq*, represents the standard deviation of the height value in the selected region, and *Max* represents the maximum height value of the region. The surface of the layers was scanned in non-contact mode, and the roughness was calculated for the largest area of 40 µm × 40 µm.

On the large area, the layer is continuous without cracks or large agglomerations. The low roughness value (Rq~11 nm) indicates a uniform and smooth surface. As can be seen from Figure 9a, the surface is nanostructured, with small grains attributed to the dopant agglomerate that form islands with sizes between 5.6 µm and 800 nm. The maximum height of the largest agglomeration is around 230 nm. These values of *Rq* (11.5 nm) and *Max* (228.1 nm) confirm the layer flatness.

To observe small details of the surface, a 2 μm × 2 μm area was scanned (Figure 9b). The nanostructured surface has small pores with sizes of 50–100 nm and small grains with sizes around 50–60 nm.

The morphology of the PbS-doped film with pores of tens of nanometers is corroborated by SEM images showing the same features.

## 4. Conclusions

PbS-doped oxide film was synthesized by the sol-gel method, spin coating technique. The PbS crystalline phase and the amorphous nature of the oxide host matrix were discussed. PbS nanoparticles with sizes ranging between 8 and 10 nm were evidenced. The band gap value determined from the PbS exciton binding energy was compared with the band gap value determined from the absorption spectrum. The different values of the bandgap energy determined using the two methods are due to the composite nature of PbS-doped film, which introduced a certain approximation of the application of Mott and Davis’ and Tauc’s laws.

The validity of the graphical method for finding *E_g_* was confirmed by the absorption spectrum fitting method showing close values for the wavelength corresponding to the band gap energy. In correlation with the absorption coefficient values, Urbach energy disclosed a certain degree of disorder specific to the composite materials.

The emission peak of the doped film is close to the emission of the precursor dopant solution, in the NIR domain, revealing that the emission feature was preserved after the deposition and drying process. The structural analysis revealed vibration modes specific to the Si-O and P-O bonds as well as to PbS nanoparticles.

The morphology of the PbS-doped film showed a relative porous network corroborated by both SEM and AFM analyses. The low roughness of the film proved a uniform and smooth surface with pores and grains of tens of nanometers.

The morphological analysis certified the presence of specific elements for the deposited film, such as Al, P, Pb, and S, together with elements O and Si specific to both the inorganic host matrix and the PbS-doped film.

## Data Availability

The datasets generated during and/or analyzed during the current study are available from the corresponding author on reasonable request.
